# Characterizing the therapeutic response to deep brain stimulation for treatment-resistant depression: a single center long-term perspective

**DOI:** 10.3389/fnint.2015.00041

**Published:** 2015-06-15

**Authors:** Andrea L. Crowell, Steven J. Garlow, Patricio Riva-Posse, Helen S. Mayberg

**Affiliations:** ^1^Department of Psychiatry and Behavioral Sciences, Emory University School of MedicineAtlanta, GA, USA; ^2^Department of Neurology, Emory University School of MedicineAtlanta, GA, USA

**Keywords:** treatment resistant depression, deep brain stimulation, subcallosal cingulate, therapeutic course, emotional reactivity

## Abstract

The number of depressed patients treated with deep brain stimulation (DBS) is relatively small. However, experience with this intervention now spans more than 10 years at some centers, with study subjects typically monitored closely. Here we describe one center’s evolving impressions regarding optimal patient selection for DBS of the subcallosal cingulate (SCC) as well as observations of short- and long-term patterns in antidepressant response and mood reactivity. A consistent time course of therapeutic response with distinct behavioral phases is observed. Early phases are characterized by changes in mood reactivity and a transient and predictable worsening in self ratings prior to stabilization of response. It is hypothesized that this characteristic recovery curve reflects the timeline of neuroplasticity in response to DBS. Further investigation of these emerging predictable psychiatric, biological, and psychosocial patterns will both improve treatment optimization and enhance understanding and recognition of meaningful DBS antidepressant effects.

## Introduction

Deep brain stimulation (DBS) is being investigated as a potential therapy for treatment-resistant depression (TRD). The DBS target with the most experience is the subcallosal cingulate white matter, alternatively referred to as subgenual or Area 25 DBS (SCC; Mayberg et al., 2005; Lozano et al., 2008, 2012; Bewernick et al., 2010; Kennedy et al., 2011; Holtzheimer et al., 2012; Puigdemont et al., 2012; Merkl et al., 2013; Ramasubbu et al., 2013). Therapeutic response has also been reported with DBS of the nucleus accumbens (NAC)/medial forebrain bundle (MFB; Schlaepfer et al., 2008, 2013; Bewernick et al., 2010, 2012), and ventral capsule/ventral striatum (VC/VS; Malone et al., 2009; Dougherty et al., 2014). While studies of DBS at these targets vary in design and rationale, small trials have demonstrated clear benefit, including long-term sustained antidepressant response (Kennedy et al., 2011; Bewernick et al., 2012; Holtzheimer et al., 2012). However, pivotal industry-sponsored trials have not demonstrated efficacy (Dougherty et al., 2014; closure of the BROADEN trial).^1^ For DBS to be validated as a reliable treatment strategy for depression, each independent approach must clearly define the precise surgical target, appropriate patient selection, time course of antidepressant response, and symptom specificity of that response. Given the small number of researchers with firsthand experience with DBS for depression, open dialog to maximize our collective experiential knowledge of this treatment is encouraged. Towards this goal, we offer a single lab perspective (Emory University, Atlanta GA, USA) from 8 years of studies of SCC DBS for TRD, highlighting the key clinical features of patient selection and the DBS-mediated therapeutic response in this target (Table 1; clinicaltrials.gov NCT00367003, NCT01984710).

**Table 1:** **Emory SCC DBS for depression experience**.

*Subjects*^1^
• 2080 potential subjects contacted • 524 completed phone screens • 274 medical records reviewed • 76 in-person assessments • 40 subjects enrolled ▪ 8 failed to meet pre-surgical depression severity criteria^2^ ▪ 1 declined surgery ▪ 1 comorbidity exclusion • *N* = 30 implanted ▪ 17 published in Holtzheimer et al. (2012) ▪ 11 manuscript in preparation ▪ 2 in current protocol
*Standardized Assessment*
• Primary outcome measure: 17 question Hamilton Depression Rating Scale (HDRS-17) ▪ Response = 50% decrease from baseline; *N* = 17/30 at one year ▪ Remission = 7 or less; *N* = 11/30 at one year
*Time Course*
• Clinical assessment weekly × 8 months, then tapered to semi-annual assessment by year 2 • 8 years since first subject enrolled • 1527 subject-months (127 patient-years)

*^1^As of 5/2015, ^2^HDRS-17 ≥20 averaged over 4 weeks prior to surgery*.

## Patients

Screening and evaluating hundreds of participants with in-depth assessments (Table 1) has shaped our view that three factors characterize a DBS responsive patient: (1) history of clear antidepressant response in early depressive episodes with evidence of inter-episode functional recovery (job, family, activities); (2) transformation from treatment-responsive to treatment-resistant depression; and (3) lack of emotional reactivity at presentation.

A typical history for those participants who respond to DBS starts with a depressive episode in their 20s that responded to antidepressant medication with symptomatic and functional recovery. In subsequent episodes, more aggressive antidepressant treatment was required and more medication failures were experienced. Most underwent electroconvulsive therapy, with an initial good response that could not be recaptured when symptoms later returned. Puigdemont et al. (2012) reported a similar pattern of disease progression. Patients’ descriptions of their depression often include themes of psychic pain, darkness, being weighted down, or being in a hole. This is accompanied by pronounced psychomotor slowing, non-fluent, monotonous speech, and limited affective range. Consequently, these qualities have taken on the most weight in our assessments of potential new subjects.

All subjects must meet a minimum severity score on a standardized rating scale for study inclusion (Table 1). However, disease severity cannot be solely defined this way. While a severity score on a depression rating scale can be informative and is important for research metrics and study integrity, assumptions about severity scores may be a confound in this chronically ill population (Bech et al., 1975; Snaith, 1977; Bagby et al., 2004). Chronicity, treatment resistance, and functional impairment define overall disease severity in addition to total symptom burden.

## DBS Lead Placement

Intraoperative testing of DBS contacts is conducted in awake subjects to explore acute stimulation effects, assess safety, and confirm electrode placement. In the SCC target, acute effects to stimulation including “lightness” and “connectedness” were initially reported and replicated in subsequent studies (Mayberg et al., 2005; Lozano et al., 2008; Holtzheimer et al., 2012; Merkl et al., 2013), but are not universal (Puigdemont et al., 2012; Ramasubbu et al., 2013). Our subjects have not experienced adverse events during intraoperative testing, although stimulation of many contacts produce no discernable behavioral effects. While the experiences reported by patients are highly personal and thus idiosyncratic, the predominant characteristic is relief from negative rather than induction of positive mood. Return of negative mood is noted soon after discontinuation of the stimulation. Acute behavioral phenomena to stimulation have been reported at other DBS targets and generally fall into categories of decreased negative mood; increased positive mood, interest, and motivation; autonomic changes including increased heart rate, sweating, or flushing; and unpleasant sensations of anxiety and mental or physical slowing (Mayberg et al., 2005; Lozano et al., 2008; Schlaepfer et al., 2008, 2013; Malone et al., 2009; Bewernick et al., 2010; Holtzheimer et al., 2012; Merkl et al., 2013; Riva-Posse et al., 2014a). Possible explanations of these phenomena include: site-specific behavioral biomarkers of antidepressant response, epiphenomena that may or may not have clinical relevance, or simply side effects of the spread of electric current to adjacent structures. In some cases, as in double vision with stimulation of the MFB (Schlaepfer et al., 2013), the specificity, predictability (based on local anatomy), and reproducibility of the effect would point toward this being a side effect. In contrast, smiling has been reported as asymmetric and time-locked to stimulation of the VC/VS target (Okun et al., 2004), but may also represent a positive affective response to the sudden absence of mental pain (Malone et al., 2009; personal observation). Such an effect would seem to blur the line between side effect and spontaneous expression of mood change.

Our observations led us to take a more systematic approach to intraoperative testing, which confirmed predictable, reproducible, contact-specific responses in most subjects (Riva-Posse et al., 2014a). The use of tractography has led us to optimize electrode placement based on the ability to engage key white matter tracts within the stimulation zone (Riva-Posse et al., 2014b). We have observed increased heart rate and skin conductance with stimulation of appropriately-positioned contacts, which have greater connectivity to the dorsal anterior cingulate and subcortical regions (Riva-Posse et al., 2014a). These autonomic effects are predictable based on the putative targets of the white matter tracts stimulated, but can tell only part of the story as additional tracts appear necessary for the antidepressant response to DBS.

The acute behavioral effects seen with intraoperative stimulation and described above are typically reproducible within the immediate post-operative period. In the days following intraoperative stimulation, it is not uncommon for our patients to experience some persisting symptom relief even though stimulation is off. This effect is strongest in the days after surgery and fades within 3 weeks. Repeating acute stimulation 1 month after surgery may reproduce the effects in attenuated form, or they may be absent. These intraoperative responses are now being evaluated as initial antidepressant effects, as biomarkers to confirm proper electrode placement, and as a probe of initial antidepressant physiological responses.

## Post-Operative Course

A 1-month post-operative recovery period, during which stimulation is off, is followed by 6 months of continuous stimulation during which no medication changes are allowed and only limited adjustments to stimulation parameters are made. In our observations, the full antidepressant response evolves through stereotypic early and late subacute phases before settling into stable long-term recovery.

### Out of the Rut …

In the first weeks of chronic stimulation, patients generally report minimal change, though outside observers notice more dynamic affect, movement, and speech. Anecdotally, family members comment that the patient “looks younger” or “is more like her old self.” Within the first month, patients report an increase in activity and may notice more things in their environment. They report little subjective change in mood, although depression ratings are usually decreasing. Patients begin to notice that they are having more emotions, including brief positive moods, before endorsing any significant or lasting lifting of their depression. Others have similarly observed that significant subjective improvement in mood may not be reported for several weeks of active stimulation (Lozano et al., 2008; Merkl et al., 2013).

Having chosen the contacts for chronic stimulation on the basis of tractography and intraoperative effect, we typically do not make changes to stimulation parameters as this clinical process unfolds. While it is not uncommon for programming changes to be required over time in neurodegenerative diseases like Parkinson’s disease, it is not clear that this level of tuning is necessary or helpful in depression (Bewernick et al., 2012; Dougherty et al., 2014). Over time, we have focused on the subtle signs of improvement, particularly with regard to a patient’s reactivity. In the absence of a clear biomarker for depression or DBS treatment effect, it is necessary to rely on clinical judgment to make such decisions. This task is more difficult in depression, where a key feature of the illness, negative mood, is not always pathological nor always attributable to major depressive disorder. With chronic stimulation, patients learn to differentiate normal negative emotions from the depressive state. However, learning to make this distinction and trust one’s ability to emerge from a sad situation appears to take time and practice and likely is affected by a patient’s premorbid personality and life experience.

### … And Into the Rough Patch

The relatively smooth and progressive improvement in depressive symptoms seen in the first weeks tends to destabilize roughly 10–12 weeks after initiation of chronic stimulation. What follows is a temporary period of subclinical emotional dysregulation characterized by increased negative affect, especially flares of anger and irritability, mood swings, and disproportionate negative emotional reactivity to environmental stressors. This is often concurrent with increased activity outside the home and increased frequency and complexity of interpersonal interactions. This period tends to last around 4 weeks. During this time, patients may report a return of depressive symptoms with increases in depression rating scores, although generally they no longer meet criteria for a major depressive episode. In the Emory experience, these fluctuations resolve without changes in stimulation parameters. At a similar time point after starting DBS, Puigdemont et al. (2012) reported a depressive recurrence in some of their patients, as well as one suicide attempt. This underscores the importance of close monitoring of patients during the first several months of DBS treatment, as this is an expected period of vulnerability. This phase may also coincide with the timing of outcome measurements in double-blind controlled clinical trials (Dougherty et al., 2014), thus confounding a normal reemergence of emotional bandwidth with return of true depressive symptoms as both load onto standard depression severity scales. Failure to establish clear distinctions between what appears to be a normal plastic process and the presumed pathological state has clear implications for evaluating efficacy and for the design of future trials. In our own studies, once this stage was recognized, parameter adjustments were halted and psychotherapy was engaged in earnest.

### Recovery Takes More than Stimulation: Chronic Response and Non-response

After 6 months of chronic stimulation, emotional hypersensitivity usually abates. Depression rating scale scores again decrease. Global Assessment of Function scores increase, as patients become more active and connected with others. They are able to imagine life further into the future and entertain longer-term goals, such as a return to employment or other productive activities. Relationships with loved ones, especially partners, change in response to the patient’s decreased dependency. Patients report an increased ability to tolerate setbacks in life and relationships. We see gradual improvement in mood and resilience through the first year and beyond, suggesting ongoing plasticity effects. Long-term follow-up studies have typically shown that the response at 1 year may continue to improve in subsequent years (Kennedy et al., 2011; Holtzheimer et al., 2012). All available evidence suggests that ongoing stimulation is necessary even in those who have been in remission for years. With device failure the euthymic state may hold for a period of time, but usually within weeks depressive symptoms gradually return. These symptoms are familiar to patients from previous episodes, although they rarely have the melancholic features characteristic of the pre-operative state. When device function is restored, therapeutic benefit returns, but it may take weeks to fully reach the previous level of wellness (Kennedy et al., 2011; Merkl et al., 2013).

Interestingly, even those considered non-responders by study criteria may nevertheless report meaningful improvement in their lives and choose to continue the treatment even when offered discontinuation and device removal, as has been the experience with other stimulation targets (Bewernick et al., 2010; Dougherty et al., 2014). In the case of SCC DBS, such a partial response may be attributable to a failure to stimulate all the white matter tracts necessary for a full response (Riva-Posse et al., 2014b). Alternatively, non-response or partial response may occur as a function of individual disease, biological, or personality characteristics. It may be that specific depressive symptom clusters that predominate in an individual may respond preferentially to stimulation of one DBS target over another. As with all psychiatric treatments, personality is bound to play a role in the nature and timing of therapeutic response or lack thereof. It is important to note that the core personality traits of patients who are accepted into DBS studies may be masked by their chronic depressive illness, such that a full understanding of their character structure can only be seen after the depressed state is lifted and the patient returns to previous behavioral patterns.

Once therapeutic contacts and parameter settings have been established, they are typically maintained over the years. Medications are generally not changed significantly once stimulation is initiated. In some instances, doses have been reduced, but generally not eliminated. However, since the protocol does not allow medication changes until after 6 months, it is possible that DBS becomes entrained with the pharmacological milieu instantiated at the time that DBS effects evolved. That said, patients do not appear to require medication to enable a DBS effect as patients on no medications can achieve clinical response and remission, although this is uncommon.

## Discussion

In our experience, patients with the best response to SCC DBS are those who have a history of treatment-responsive depressive episodes with good inter-episode recovery, but undergo a malignant transformation and no longer respond to standard therapies. The antidepressant response to SCC DBS may be best described in acute, subacute, and chronic phases. Acute stimulation of appropriately positioned electrodes is frequently associated with feelings of relief or increased awareness. These responses are specific to each individual and highly reproducible with repeated testing in the intraoperative and perioperative period.

That said, with chronic stimulation, initial changes are noticed first by others. Patients notice increased activity and become more aware of their environment before they notice improved mood. As patients experience more sustained improvement in mood and more critically, increased emotional range, they move from a state of relative stability around a low negative to relative instability, with heightened emotional sensitivity and reactivity. Close follow-up and reassurance during this period of emotional recalibration is warranted, though frequent stimulation parameter adjustments are not. This intermediate stage of recovery, generally lasting several weeks, gives way to increased stability and resilience manifest by progressive improvement in depressive symptoms that are maintained over months and years. While the steepest improvement tends to be seen in the first 6 months, DBS responders report continued gradual improvement over one or more years of treatment. Discontinuation of stimulation during this recovery phase nonetheless leads to a gradual return of symptoms over several weeks. Current studies are exploring how rehabilitative strategies may best synergize with these distinct phases of recovery.

The gradual and predictably bumpy recovery curve in SCC DBS for depression stands in contrast to the response to DBS observed in Parkinson’s disease and essential tremor, where the effect is apparent immediately, maximal effect is reached within hours, and with discontinuation, primary symptoms return immediately (Hristova et al., 2000). The response to DBS for dystonia is more similar to that of depression, as it develops gradually and maximum effect is seen only after months of stimulation (Yianni et al., 2003). In dystonia and depression, neuroplasticity and CNS remodeling may be critical to the long-term treatment response to DBS (Ruge et al., 2011; Gibson et al., 2014). Indeed, changes in serum levels of brain-derived neurotrophic factor are abnormally low and increase with antidepressant treatment (Brunoni et al., 2008, meta-analysis) and in response to chronic DBS in an animal model (Hamani et al., 2012). DBS-induced plasticity may allow for changes in regional and network activity that ultimately result in a normalization of depression-related pathology. PET scans 3 and 6 months following DBS implantation show activity changes in depression-relevant regions, including normalization of hyperactivity in the SCC (Mayberg et al., 2005; Lozano et al., 2008; Bewernick et al., 2010). Prior to PET scan changes, autonomic changes accompany the behavioral response to acute stimulation (Riva-Posse et al., 2014a), and changes in EEG frontal theta concordance after 1 month of stimulation predict 6 month response to DBS therapy (Broadway et al., 2012). The time course varies for different regions, which may reflect both direct and indirect actions on these networks by acute and chronic stimulation. Comparable findings have been demonstrated using EEG, suggesting a differential time course of changes with long-term stimulation. These findings are consistent with the clinical observations of phase response characteristics and further they work toward understanding the mechanism of DBS treatment.

It is hoped that knowledge gained from these small, open-label, mechanistic investigations will inform the design of larger scale efficacy trials for DBS. Those who would design such trials face significant challenges. The heterogeneity of depression may be obscuring subsets of patients who are the most appropriate candidates for DBS at each anatomical target. Inability to effectively quantify the desired patient characteristics creates a problem for clinical trials, where everything must be operationalized. Allowances must be made in treatment decision algorithms for discrepancies between clinical impression and standardized rating scores. During the transient period of emotional hypersensitivity in the subacute phase, depression ratings may worsen, which may trigger protocol-defined parameter changes that interrupt the natural course of recovery thus confusing the clinical picture and ultimately, the demonstration of efficacy. Development of next-generation closed-loop DBS systems that are capable of monitoring and responding to changes in neuronal signals may further improve the conduct of future clinical trials (Afshar et al., 2013; Hosain et al., 2014; Smart et al., 2015). Such systems will be critical to identifying biomarkers of DBS-mediated antidepressant response and hence guide treatment optimization.

## Conflict of Interest Statement

Dr. Crowell has received funding from the American Psychiatric Association’s Psychiatric Research Fellowship Award funded by Eli Lilly and Company and the National Institutes of Health Loan Repayment Program. Dr. Mayberg has received funding from the Dana Foundation, Woodruff Fund, Stanley Medical Research Institute, and the Hope for Depression Research Foundation and consulting and intellectual licensing fees from St. Jude Medical Inc. All other authors report no commercial or financial relationships that could be construed as a potential conflict of interest.

## Footnotes

^1^http://www.sjm.com/broaden
